# Direct costs associated with the appropriateness of hospital stay in elderly population

**DOI:** 10.1186/1472-6963-9-151

**Published:** 2009-08-22

**Authors:** Joaquín F Mould-Quevedo, Carmen García-Peña, Iris Contreras-Hernández, Teresa Juárez-Cedillo, Claudia Espinel-Bermúdez, Gabriela Morales-Cisneros, Sergio Sánchez-García

**Affiliations:** 1 Departamento de Negocios Internacionales, Instituto Tecnológico y de Estudios Superiores de Monterrey, México, D.F., México; 2 Unidad de Investigación Epidemiológica y en Servicios de Salud, Área Envejecimiento, Centro Médico Nacional Siglo XXI, Instituto Mexicano del Seguro Social, México, D.F., México; 3 Unidad de Investigación en Economía de la Salud, Centro Médico Nacional Siglo XXI, Instituto Mexicano del Seguro Social, México, D.F., México; 4 Subdirección de Información de Recursos Humanos y Materiales, Dirección General de Información en Salud, Secretaria de Salud, México, D.F., México

## Abstract

**Background:**

Ageing of Mexican population implies greater demand of hospital services. Nevertheless, the available resources are used inadequately. In this study, the direct medical costs associated with the appropriateness of elderly populations hospital stay are estimated.

**Methods:**

Appropriateness of hospital stay was evaluated with the Appropriateness Evaluation Protocol (AEP). Direct medical costs associated with hospital stay under the third-party payer's institutional perspective were estimated, using as information source the clinical files of 60 years of age and older patients, hospitalized during year 2004 in a Regional Hospital from the Mexican Social Security Institute (IMSS), in Mexico City.

**Results:**

The sample consisted of 724 clinical files, with a mean of 5.3 days (95% CI = 4.9–5.8) of hospital stay, of which 12.4% (n = 90) were classified with at least one inappropriate patient day, with a mean of 2.2 days (95% CI = 1.6 – 2.7). The main cause of inappropriateness days was the inexistence of a diagnostic and/or treatment plan, 98.9% (n = 89). The mean cost for an appropriate hospitalization per patient resulted in US$1,497.2 (95% CI = US$323.2 – US$4,931.4), while the corresponding mean cost for an inappropriate hospitalization per patient resulted in US$2,323.3 (95% CI = US$471.7 – US$6,198.3), (p < 0.001).

**Conclusion:**

Elderly patients who were inappropriately hospitalized had a higher rate of inappropriate patient days. The average of inappropriate patient days cost is considerably higher than appropriate days. In this study, inappropriate hospital-stay causes could be attributable to physicians and current organizational management.

## Background

Ageing of the Mexican population is one of the higher impact phenomena which began manifesting during the XX Century, and which will undoubtedly be an essential element in the creation of the history of Mexico in the XXI Century. Life expectancy of Mexicans doubled during the second half of the last century, raising from 36 years in 1950 to 74 years in year 2000, and it is expected to reach 80 years in 2050 [1].

Ageing of the population will imply a greater demand of health services, as a consequence of the high rates associated morbidity. Among the diseases of the elderly population are chronic-degenerative conditions, that make them fragile and turns them into a population that utilize many health services. However, it is well known that part of the hospital resources are used inadequately, either because the patients receive assistance that does not turn into health benefits or because care services could be provided at different institutional levels representing lower costs [2,3].

Evaluation of resource use in hospital health systems allows to establish the necessary actions which will correct the identified organizational problems. Unjustified hospital admissions and stays of elderly patients, do not only increase costs but are also related to poor health service and higher mortality resulting from several complications that come together, for instance, hospital infections, pressure ulcers or venous thrombosis, among others. Thus, the permanent evaluation of hospital service utilization is an essential topic that must be considered to improve resource assignment and to increase the quality of medical assistance in institutions that render services [4,5].

There are several reports that have calculated inappropriate utilization figures. However, very few have studied the associated factors [6-8].

Finally, the economic analysis of health services provided to the elderly population was done in general terms by other researchers [9,10], concluding that the associated costs including medical assistance required by the elderly are higher in comparison to the rest of the population. Then again, the cost of appropriate and inappropriate hospital stay in the elderly population has not been identified, at least in developing countries where medical practice vary and could have important effects on the hospital budgets in comparison with other developed countries. Even if medical costs would be expected to be lower in developing countries this expenses could have a significant impact over health budgets. Previous studies of inappropriate hospital length of stay showed that this phenomenon is mainly generated by doctors whom are not required to justify individual hospital stay, and hence there is an incentive to prolong hospitalization when there are empty beds [11,12]. In addition, other researchers identified a number of key causes of inappropriate admissions and lengths of stay, including: the limited capacity of health and social care resources; poor communication between primary and secondary care clinicians and the cautiousness of clinicians who manage patients in community settings [13]. In this sense, economic literature have been elaborated in order to estimate direct medical costs of length of stay and to estimate budget impact of inappropriate hospitalizations [14,15]. The aim of this study was to estimate direct medical costs associated with inappropriateness hospitalizations days in the elderly population within a representative Mexican hospital.

## Methods

A retrospective study was conducted, reviewing clinical files of 60 years of age and older patients (n = 7,540) admitted from January 1st to December 31st of 2004 within the Mexican Social Security Institute (IMSS).

The IMSS was created by law in 1943 and is funded by the government, employers, and employees. It is a social security system; therefore, the only requirement to be registered is to be employed, regardless of one's state of health. Workers, their parents, and other close relatives are assigned to a Family Medicine Unit, which is the primary health care provider. The IMSS offers a comprehensive package of benefits that include health care services at all levels of care and economic benefits such as a pension. Medical services are provided by levels of care and a reference system. Mexico City has a population of nearly 860,000 adults aged 60 years and older, 418,000 of whom (48.6%) are affiliated to the IMSS. IMSS has 16 general hospitals in Mexico City. From those, the General Hospital "Dr. Carlos Mac Gregor Sánchez Navarro" was selected. This hospital is one of the biggest hospitals in Mexico City and it has 278 total beds. A total of 465 physicians and 705 nurses work there. General services as internal medicine, surgery, intensive care, pediatrics, plus some specialized services such as hematology, nephrology integrate the services provided.

The size of the sample was estimated assuming that 10% of the hospital admissions are inappropriate [16], with an accuracy of 0.02 and a reliability level of 95%. The required sample was of 863 files, plus a 20% considering the exclusion criteria. The files were selected simply by random.

We excluded files of patients with voluntary hospital discharges, patients with illegible files, lacking information necessary for evaluation, files of patients transferred to other hospitals and those that were not found when the information was collected.

The unit of analysis was made up of the admission process clinical hospital file registries, first days and subsequent patient days to admission, with the exception of the medical discharge day. The version adapted to Spanish and validated in the elderly population by our Appropriateness Evaluation Protocol (AEP) group was used as a tool to determine hospitalization appropriateness of the study subjects [17-19]. The AEP was previously validated in elderly Mexican population in 2005 [16]. The first AEP set comprised 16 criteria that establish the need for hospitalization on the hospital admission day. The first ten criteria were related with the severity of the patient's clinical condition, while the remaining six criteria were associated with frequency with which the provided health services were utilized. The presence of at least one of these criteria on the first admission day was enough to consider it appropriate; on the other hand, it was considered inappropriate when it did not comply with any of the criteria. In addition, to determine the need for further days of stay subsequent to the admission day -with the exception of the admission day itself-, we used a second AEP set comprising 27 criteria related with medical service rendered, nursing services and the patient's clinical condition. As in the previous case, compliance with one sole criterion was sufficient to consider the patient days reviewed as appropriate and inappropriate when it did not comply with any of the criteria. Likewise, admission causes and inappropriate patient days were reported.

A group of three nurses with bachelor degrees was previously trained in AEP handling and application. The intra-reviewer agreement of hospital admissions presented a Kappa coefficient of >80%, while patient days yielded Kappa>86%. Regarding to inter-reviewer agreement, the result was Kappa>93%, and >85% for admission and patient days, respectively.

Age and gender were also collected as well as admission service and comorbidity. Comorbidity was defined as the presence of one or more disorders (or diseases) in addition to a primary disease or disorder.

The economic analysis consisted of an estimate of direct medical costs associated with appropriate and inappropriate assistance provided to the subjects included in the study during their hospital stay. Direct medical costs include all related costs generated during the hospitalization of patients (drugs, laboratory and radiologic exams, inter-visits to other specialists, procedures, emergency and administrative expenses). Neither out-of-pocket expenses nor indirect costs (productivity losses) are included since the perspective is from the third-party payer's.

The economic analysis procedure was realized by resource identification, measurements and valuation. Identification and measurement of the resources was carried out through a review of the clinical files to generate a listing of goods and services used in every hospital stay, identifying the headings corresponding to patient days, surgeries, special procedures, laboratory tests and office examinations, medication and inter-visits to other specialists.

Cost estimates were obtained applying the unitary costs of goods and services used in every hospital stay, which were obtained from IMSS institutional data base, accessing  web page, as well as from the notices of IMSS Planning and Finance Division, 2006. The costs are expressed in US Dollars according to the 2006 exchange rate officially reported by Banco de México. No discount rate was applied, since the analysis horizon was within one year.

### Statistical Analysis

Of the total of identified hospital stays, values of the mean of the interest socio-demographic and clinical variables were obtained, and the cases corresponding to sex, age range, service to which the patient was admitted, appropriate admission, length of stay (in days) and number of comorbidities conditions were determined. Similarly, the reasons why the admission was considered as inappropriate and patient days were identified and estimated. The ratio of inappropriate days refers to the number of inappropriate length of stay per patient measured in days divided by the number of total patient days (appropriate plus inappropriate) by 100.

Mean inappropriate days only considering patients who presented inappropriate days.

Regarding the patient days: direct medical costs per patient with their reliability intervals were estimated at 95% (95% CI). Mean costs of appropriate and inappropriate patient days were calculated from the mean cost for each group (patients). In addition, the mean costs were compared through the t-student test for independent samples, with a 95% significance level.

Finally, a multivariate analysis was performed to find out what impact do socio-demographic factors (sex and age) and clinical factors (appropriate admission, length of stay, number of comorbidities conditions and inappropriate patient days) have on the hospital stay's total cost.

The patient days cost was transformed to a natural logarithm in the multivariate analysis to reduce the variability shown by costs estimated for patients with different pathologies and several severity levels: The latter result in heterogeneous patient days and different quantities of hospital resource use. Sex and admission variables were handled as dichotomies, while age, length of stay, number of comorbidity conditions and inappropriate days variables were handled as a continuum. Variables used in the multivariate analysis were chosen from the available factors founded in the hospital clinical files for each patient.

The Ordinary Least Squares assumption evaluation was done paying special attention to the homoscedasticity (assessed with the Breusch-Pagan and White tests), as the transversal cohort of patients included in the sample presented atypical factors, such as hospital stays that lasted more than 90 days and variation in the use of resources resulting from the diversity of reasons for medical assistance.

### Ethics

The research protocol for this study (2004-3607-0009) was reviewed and approved by the Local Commission of Health Research and the Sub-Committee of Ethics of the Mexican Social Security Institute, Delegation 3 and 4 in Mexico City.

## Results

### General Characteristics

A total of 1,036 hospital clinical files were selected, of which 31.2% (n = 312) were excluded given that they did not fulfill the age or the admission period criteria. The final sample comprised 724 files: 51.9% females with an average age of 76.9 (± 9.2) years and the remaining 48.1% males with an average age of 73.9 (± 8.0) years.

### Inappropriate Admission and Hospital Stays

The patient days mean was 5.3 (95% CI = 4.9–5.8) days, with 5.6 (95% CI = 4.9–6.3) and 5.0 (95% CI = 4.6–5.5) days for females and males, respectively.

Of the 724 hospital admissions, only 1.5% (n = 11) were classified as inappropriate. Those admitted inappropriately had more inappropriate patient days (17.1%), in comparison to those whose admissions were appropriate (5.0%). Among the 1.5% patients admitted inappropriately, who only required nurse assistance were 63.6% (n = 7). On the other hand, 27.3% (n = 3) of the patients required assistance from a hospital specialized in chronic diseases; and premature admission of one day or more previous to appointments for tests resulted in 9.1% (n = 1).

From the 724 files reviewed, 12.4% (n = 90) were classified with at least one day of inappropriate hospital stay. The mean of inappropriate patient days per patient of the 90 files was 2.2 days (95% CI = 1.6 – 2.7). The reasons for considering them as inappropriate stays were that there was no diagnostic plan and/or treatment, 98.9% (n = 89); that there was a planned discharge without written orders, 3.3% (n = 3); there was no work at the hospital those days (certain diagnostic procedures are not performed during the weekend or in holidays), 2.2% (n = 2); patient programmed for diagnostic tests or treatment (including surgery) whose appointment was cancelled due to any other reason (for instance, an emergency case is put before an elective case or essential personnel from the hospital was ill, etc.) 1.1% (n = 1); and, others 2.2% (n = 2). It is worth pointing out that five files fulfilled more than one of these causes.

Five percent (5.1%, n = 198) of the 3891 days of hospital stays were classified as inappropriate. Table 1 shows the ratio of inappropriate patient days according to the characteristic of the sample.

**Table 1:** Appropriate of admission and patient days.

	**Patients**	**Patient days**	**Rate of inappropriateness days**
	**n (%)**	**n (%)**	**Rate (95% CI)**
**Total**	724 (100)	3891 (100)	5.1 (3.4 – 5.9)
			
**Sex**			
Female	376 (51.9)	2118 (54.4)	5.0 (3.2 – 5.5)
Male	348 (48.1)	1773 (45.6)	5.2 (3–5 – 6.1)
**Age (years)**			
60–64	87 (12.0)	446 (11.5)	5.2 (3.3 – 5.8)
65–69	117 (16.2)	590 (15.2)	5.6 (3.8 – 6.1)
70–74	137 (18.9)	635 (16.3)	6.3 (4.6 – 6.9)
75–79	137 (18.9)	719 (18.5)	7.0 (5.6 – 7.4)
80–84	119 (16.4)	717 (18.4)	2.4 (1.3 – 3.2)
≥ 85	127 (17.5)	784 (20.1)	4.5 (2.5 – 5.4)
**Service admitted to:**			
Surgery	126 (17.4)	438 (11.3)	8.4 (6.5 – 9.2)
Internal medicine	598 (82.6)	3453 (88.7)	4.7 (4.1 – 5.3)
**Appropriateness admission**			
Yes	713 (98.5)	3856 (99.1)	5.0 (4.3 – 6.4)
No	11 (1.5)	35 (0.9)	17.1 (6.3 – 23.2)
**Length of stay (days)**			
1–2	213 (29.4)	320 (8.2)	2.2 (1.4 – 2.8)
3–4	200 (27.6)	687 (17.7)	5.7 (4.8 – 6.5)
5–6	131 (18.1)	717 (18.4)	4.0 (3.4 – 4.4)
7–8	69 (9.5)	505 (13.0)	4.0 (3.2 – 4.7)
9–10	33 (4.6)	313 (8.0)	4.8 (4.0 – 5.5)
≥ 11	78 (10.8)	1349 (34.7)	6.5 (6.1 – 6.9)
**Number of comorbidities conditions**			
0	125 (17.3)	488 (12.5)	5.3 (4.7 – 5.8)
1	71 (9.8)	385 (9.9)	9.4 (8.1 – 11.3)
2	169 (23.3)	938 (24.1)	5.1 (4.3 – 5.8)
3	174 (24.0)	992 (25.5)	3.1 (2.8 – 3.6)
≥4	185 (25.6)	1088 (28.0)	5.2 (4.9 – 6.3)

### Direct medical costs and associated factors

Mean costs of inappropriate hospital days was calculated from the mean cost of 90 patients (files) founded in our sample. The mean cost for an appropriate hospitalization per patient resulted in US$1,497.2 (95% CI = US$323.2 – US$4,931.4), while the corresponding mean cost for an inappropriate hospitalization per patient resulted in US$2,323.3 (95% CI = US$471.7 – US$6,198.3), showing statistically significance difference among them (p < 0.001). Differences are mainly explain due to the higher number of unnecessary days the patient is treated in the hospital using healthcare resources such as additional laboratory and gabinet exams (7%), inter-visits to other specialists (28%), drugs (5%) and administrative expenses (60%). Nevertheless, an inappropriate day costs 18% less than an appropriate day due is less intensive in resource use (patients are mainly in observation not treated intensively).

Table 2 shows the mean cost of hospital stay according to the sample's characteristics. When a comparison was made between the total cost means of appropriate and inappropriate stays, we noticed that there was no significant statistical difference in 70–74 year olds, 80–84 year olds and 85 or more year olds. Likewise, no significant differences were found between cost means of appropriate and inappropriate stays of elderly patients who were admitted for surgery services. Regarding appropriate admissions of elderly patients, a difference between means (p < 0.001) was found.

**Table 2:** Cost of hospital stay in the studied elderly patient sample

	**Cost of hospital stay**				
	**Appropriate**		**Inappropriate**		
	**Mean**	**(95% CI)**	**Mean**	**(95% CI)**	**p**
**Sex**					
Female	1,555.6	(319.4 – 5,413.1)	2,528.0	(665.7 – 5,817.9)	p < 0.001
Male	1,432.5	(328.4 – 4,459.5)	2,132.0	(470.6 – 6,012.7)	p < 0.001
**Age (years)**					
60–64	1,409.4	(317.2 – 3,762.7)	2,183.2	(1,031.0 – 4,789.2)	p = 0.018
65–69	1,261.6	(314.3 – 3,105.3)	3,374.0	(481.0 – 16,991.7)	p = 0.001
70–74	1,397.5	(336.6 – 4,234.4)	1,703.3	(475.8 – 3,582.2)	p = 0.239
75–79	1,504.2	(327.5 – 5,658.4)	2,393.7	(598.0 – 7,413.3)	p = 0.010
80–84	1,679.5	(299.8 – 5,665.7)	2,395.0	(835.4 – 5,451.9)	p = 0.116
≥ 85	1,696.3	(394.6 – 4,618.6)	2,283.6	(1,002.3 – 3,887.6)	p = 0.062
**Service admitted to:**					
Surgery	1,191.8	(334.3 – 5,059.3)	1,611.3	(362.1 – 3,596.7)	p = 0.197
Internal medicine	1,536.0	(317.8 – 4,871.3)	2,456.2	(655.0 – 6,594.5)	p < 0.001
**Appropriateness admission**					
Yes	1,497.2	(323.2 – 4,931.4)	2,278.0	(763.6 – 5,942.1)	p < 0.001
No	NA		2,515.3	(363.7 – 14,772.5)	NA
**Length of stay (days)**					
1–2	683.0	(202.4 – 1,623.6)	365.8	(355.9 – 375.6)	p = 0.179
3–4	1,016.3	(512.2 – 1,978.7)	1,239.5	(476.1 – 2,855.7)	p = 0.020
5–6	1,548.3	(835.4 – 2,466.1)	1,403.0	(939.8. – 2,169.4)	p = 0.210
7–8	2,019.1	(1,195.4 – 2,991.3)	1,867.8	(1,181.6. – 3,145.1)	p = 0.350
9–10	2,351.6	(1,546.6 – 3,197.5)	2,398.4	(1,554.1 – 3,171.2)	p = 0.813
≥ 11	4,141.7	(2,055.5 – 7,246.8)	4,490.7	(1,935.5 – 14,042.9)	p = 0.562
**Number of comorbidities conditions**					
0	1,486.8	(320.6 – 4,883.8)	2,266.0	(585.1 – 6,778.6)	p = 0.038
1	1,369.7	(334.3 – 3,002.8)	2,425.8	(1,052.4 – 3,605.6)	p < 0.001
2	1,413.8	(332.2 – 4,115.5)	1,943.6	(684.3 – 4,110.3)	p = 0.010
3	1,659.6	(346.3 – 6,202.7)	2,900.7	(576.6 – 12,091.9)	p = 0.005
≥ 4	1,477.1	(282.4 – 4,272.3)	2,051.4	(600.7–5,048.5)	p = 0.106

**NA **= Not Apply

On the subject of patient days, those who remained hospitalized for three to four days showed a significant difference (p = 0.020) according to the appropriate-stay criterion. In elderly patients who showed four or mores comorbidities conditions, no significant differences were found.

The multivariate estimation by the Ordinary Least Squares model showed heterocedasticity problems (Breusch-Pagan and White tests with p < 0.001), therefore Generalized Least Squares were applied. The latter involves a regression model that is worthy to identify significant associated factors of hospitalization costs. As a dependent variable the hospitalization costs (previously transformed using the logarithm method) was used and on the right side, independent variables included in the regression model were: sex, age, inappropriate admission (0 = appropriate, 1 = inappropriate), total length of stay (appropriate + inappropriate days), comorbidities and the number of inappropriate days only. Results are shown in Table 3. The significant variables (p < 0.05) obtained through this model were, the intercept, age, inappropriate-patient days and the total number of patient days. As was expected, the latter had the greatest impact on the hospital-stay costs. Therefore, the multivariate regression showed that the hospitalization costs are highly associated with the age, total length of stay and the inappropriate length of stay.

**Table 3:** Generalized Least Square model using as dependent variable the transformed logarithm of hospital costs (n = 724)

**Variable**	**β (SD)**	**p-value**
Intercept	-135.0 (3.3)	<0.001
Sex	12.4 (39.5)	0.712
Age	4.9 (2.2)	0.005
Inappropriateness admission	-135.0 (223.1)	0.075
Patient days	222.2 (3.7)	<0.001
Comorbidities	7.7 (14.0)	0.708
Inappropriateness days	-39.7 (12.8)	0.001

**β = **Parameter estimate for each variable considered in the GLS model

**SD = **Standard Deviation

## Discussion

This study reported a 12% rate of inappropriate hospitalization use, which falls within the 5% to 74% [18,20-26] ranges reported in literature.

It is worth to mention that this study has methodological weaknesses derived from the retrospective review of the clinical files and from the quality of its design; consequently it is possible that insufficient or incomplete information could generates estimate biases. Overall resource use could be underestimated and consequently, hospital stays costs could be affected [27,28].

On the other hand the study presents a selection bias related to the design of the study. Due to feasibility, those who were not admitted to the hospital were not included. Consequently the rate of false negative could be underestimated. A second stage of this study could be to test the AEP criteria in the emergencies room [16].

In spite of its weaknesses, this study is not based on certain assumptions, such as that the assistance services are always appropriate from the viewpoint of the specialist doctor who renders them, or that some socio-economic factors and clinical circumstances affect the illness' evolution, justifying hospital admission [16,27]. To overcome these circumstances, the AEP instrument was used to collect the information. This instrument is characterized by its high reliability and adequate validity to identify inappropriate hospital use [16,17].

In our study, the cause to classify hospital stays as inappropriate were attributable to the specialist doctor and/or organizational type, and could be solved by implementing interventions in the hospital assistance process, as reported in Sweden. In a study carried out at the internal medicine services of a university hospital, 15% of hospital stays were considered as inappropriate. In response to this, an intervention was implemented in the hospital assistance process resulting in a reduction of up to 9% of inappropriate stays [29].

Nevertheless, an important element to consider in the design of any strategy of this type is that patient days necessary for the recovery of the patient are difficult to determine. We must bear in mind that response to treatment is different in each individual and is frequently conditioned by the severity of the illness and its evolution time (which to begin with, led to the problem that caused the hospitalization), the patient's age (which in a certain way represents the individual's biological reserve), the presence and severity of comorbidities, timely medical assistance, psychological and emotional condition of the patient, as well as support of the social networks the patient has available [30,31].

In our study it was possible to identify that elderly patients admitted inappropriately had more inappropriate patient days (17.1%), in comparison to those whose admissions were appropriate (5.0%). Regarding costs, inappropriate stays were 55.2% higher than the appropriate stays. The frequency of inappropriate admissions was lower than we expected. This result can be due to the fact that a hospital with a high demand for services and with low resources, such as the hospital in which the study was carried out, would render evaluation of the pertinence of the hospital admission of an individual more rigorous. This was not a private hospital and/or one with private medical insurance so it strengthens the possibility that the hospital admissions and stays increases in those who really do need it. However, the study presents a selection bias related to the design of the study. Due to feasibility, those who were not admitted to the hospital were not included and the rate of false negative could be underestimated. Consequently our study included only appropriately and inappropriately admitted patients. A second stage of this study must be to evaluate the AEP criteria in the emergencies room to be able to evaluate patients inappropriate and appropriate non-admitted patients to the hospitalization areas.

Heterocedasticity problems of the Ordinary Least Squares regression were mainly due to the heterogeneous characteristics of the patients and the diversity of resources used when the patient days number increased. The dependent variable was transformed applying the natural logarithm, process which did not stabilize the variance. Accordingly, a Generalized Least Squares model was used to correct said heterocedasticity problems. The results show that age, number of patient days and inappropriate patient days are variables that have an effect on hospital-stay costs. Hence it was identified that every year of the patient's life tends to increase total hospitalization costs to US$4.9, while every appropriate patient day raises them close to US$222.2.

On the other hand, the daily cost of inappropriate hospital-stay increases the total hospitalization cost to US$182.3. This states that daily inappropriate costs are 18% under daily appropriate costs due a lower intensive medical care. Therefore, this inappropriate patient day cost reflects the fact that, in view of a lack of an additional diagnostic and/or treatment plan in the clinical file, all the resources for patient assistance are not used as in the case of appropriate hospital-stay. This includes also other resources such as laboratory tests and office examinations, as well as inter-visits to other specialists.

This evaluation identifies that there is an inappropriate and unnecessary use of hospital service resources provided to the elderly patients. In other words, a variety of human and technical resources and infrastructure are used in circumstances were they are not indicated form a strictly medical viewpoint, leading to a raise of hospital-stay costs. Authors recommend that in the future this type of studies should be carried out for elderly patients with specific diseases.

The elderly population has been identified as the greatest consumer of health services, generating expenses which mainly arise from their hospitalization in medical centers [32]. Health Systems around the world are facing very similar challenges. Particularly in Latin America, where the demographic transition has been too fast, the increasing demand of health services by the elderly is a common issue.

It is well known that hospital resources are not used in a proper way. In some occasions, elderly patients receive services that they do not really need. Others, benefits received are not significant or the care given at the hospital could be given through other health care schemes less expensive and more appropriate such as nurses at home, day care hospitals and so on [33,34]. In that sense, our results are of interest for countries with similar social security systems. Mexico, as many other developing countries, has to move on to different organization models. Consequently, even when costs obviously can not be generalized, the whole sense of the paper can be of important for other regions.

Therefore, an improvement of elderly patient's assistance and an optimum utilization of medical assistance resources, especially of the hospitalization services, represent an important challenge for health service providers worldwide.

## Conclusion

Elderly patients who were inappropriately hospitalized had a higher rate of inappropriate patient days. The average of inappropriate patient days cost is considerably higher than appropriate days. In this study, inappropriate hospital-stay causes could be attributable to physicians and current organizational management.

## Competing interests

The authors declare that they have no competing interests.

## Authors' contributions

JFM-Q originated the idea for this study, did the research proposal, data analysis, and prepared the manuscript. CG-P, IC-H, and TJ-C contributed to the research proposal, reviewed the analysis, and participated in the preparation of the manuscript. CE-B participated in the interpretation of the data and in the discussion of the paper. GM-C participated in the research proposal and reviewed the manuscript. SS-G designed and conducted the original proposal and was involved in the data analysis and in the preparation and discussion of the manuscript. All authors read and approved the final manuscript.

## Pre-publication history

The pre-publication history for this paper can be accessed here:


